# Construction of Zeolite-Loaded Fluorescent Supramolecular on-off Probes for Corrosion Detection Based on a Cation Exchange Mechanism

**DOI:** 10.3390/nano11010169

**Published:** 2021-01-11

**Authors:** Jing Lv, Qing-Xian Yue, Rui Ding, Qi Han, Xin Liu, Jia-Long Liu, Hui-Jie Yu, Kang An, Hai-Bin Yu, Xiao-Dong Zhao

**Affiliations:** 1 School of Ocean, Yantai University, Yantai 264005, China; lvjing330@163.com (J.L.); y9482xian@163.com (Q.-X.Y.); hanqi_2000@163.com (Q.H.); m17861102576@163.com (X.L.); liujialong2021@163.com (J.-L.L.); Yuhj90@126.com (H.-J.Y.); ak13869182876@163.com (K.A.); 2 Key Laboratory of Marine Materials and Related Technologies, Chinese Academy of Sciences, Zhejiang Key Laboratory of Marine Materials and Protective Technologies, Ningbo 315201, China; haibinyu@nimte.ac.cn

**Keywords:** corrosion detection, chemical probe, zeolite, fluorescence

## Abstract

Metal engineering structures are commonly covered and protected by coatings. However, the early local corrosion under the coatings and at defects is difficult to detect and discover. Visibility to the naked eye means that corrosion has already developed and expanded. Therefore, it is practical significant to detect the early corrosion of coated metal. Based on the formation of iron ions and anodic acidification in the local corrosion process, iron ions and proton responsive fluorescent rhodamine B acylhydrazone on-off probes are prepared by newly improved methods and denoted as RBA. RBA are loaded on the surface and in the lattice cage of zeolite (ZEO) to protect RBA from premature exposure to the corrosive environment and fluorescence quenching. In corrosive environments, the RBA loaded on the surface are released and complex with iron ions in the environment to activate fluorescence characteristics. Simultaneously, due to the cation exchange of ZEO, iron ions enter the lattice cage of ZEO and combine with RBA in the lattice cage to turn on fluorescence. When applied in epoxy coatings, the RBA/ZEO effectively indicate the occurrence of corrosion under the coatings and at defects, and accurately locate the corrosion site. Nano-scale ZEO (or RBA/ZEO) fill the micropores such as pinholes and defects of the coatings, and increase the difficulty of diffusion and penetration of corrosive media into the coatings. The application of RBA/ZEO functional filler not only do not weaken the main anti-corrosion performance of the coatings, but also significantly improve it.

## 1. Introduction

Organic anti-corrosion coatings were widely used because they were cheap and convenient corrosion protection methods. However, when corrosive media penetrated the coatings or small defects (scratches or impurities, etc.) appeared in the coatings, local corrosion occurred. The medium penetration region or coating defects acted as anode, and their peripheral area was the cathode. The corrosion was accelerated by the small anode and large cathode structure. Due to the coatings, this kind of corrosion was concealed. Visibility to the naked eye meant that corrosion has already developed and expanded. Therefore, timely detection of local corrosion that occurs under the coatings and in the defect is very important. Driven by reality, abundant detection technologies were developed, such as boron mirrors for tank inner walls [1], magnetic flux leakage technology for pipeline and tank corrosion [2], CCD imaging methods [1], ultrasonic guided waves technology [3,4], X-ray imaging methods [5], infrared thermal imaging methods [6], electromagnetic sensor [7,8], electrochemical probe technology, etc. [9]. These technologies performed well in specific fields, but faced various limitations. For example, insufficient sensitivity, radiological hazard, inconsistent large-throughput scanning and precise positioning, and inadaptability to small spaces. Electrochemical methods usually need to be equipped with corresponding probes for different corrosion environments and corrosion types [10]. The complexity of the technologies and the inconvenience of implementation made them extremely difficult and expensive to detect local corrosion under the coatings and in the defects in time. Corrosion factor-responsive fluorescent molecular switches are potentially effective methods and a research hot spot in this field. The local corrosion of metals is accompanied by the generation of metal ions and local acidification [11]. Therefore, changes in fluorescence behavior caused by the influence of metal ions and hydrogen ions theoretically allow the detection of local corrosion [11,12]. Based on this concept, some researchers have developed hydrogen ion-responsive modified coumarin imidazole and aluminum ion-responsive fluorescent protein derivatives and Morin fluorescent agents for corrosion detection of aluminum alloy [12,13]. The complexation of 8-hydroxyquinoline with ${\mathrm{Fe}}^{3+}$ or ${\mathrm{Al}}^{3+}$ enhanced the fluorescence, which made it possible to theoretically detect the corrosion of steel and aluminum alloy, and also indicated the presence of interferences [14]. Some ${\mathrm{Fe}}^{3+}$-selective fluorescent molecules were discovered and developed, such as ferrichrome analog and desferrioxamine B derivatives. Due to the paramagnetic effect caused by the unpaired d electrons in ${\mathrm{Fe}}^{3+}$, which promotes the energy dissipation of the excited state in the non-radiation process, these ${\mathrm{Fe}}^{3+}$-sensitive fluorescent molecules are based on the fluorescence quenching mechanism. For the convenience and significance of observation, a ${\mathrm{Fe}}^{3+}$-responsive fluorescent probe based on the fluorescence ‘turn on’ mechanism is a more suitable choice. The fluorophores of rhodamine and its derivatives display excellent photophysical properties and have been widely used to design cation-selective fluorescent probes. The fluorescence activity and intensity of rhodamine B increases significantly in acidic or ${\mathrm{Fe}}^{3+}$ environments [15].

Organic anticorrosion coatings are heterogeneous and complex in terms of chemical composition, macro-/microstructure, surface and interface properties. Moreover, the corrosive environment contains a wide variety of ions. The direct doping of fluorescent probes could lead to premature exposure to the complex coating environment. Fluorescent probes are easily affected by coating components or infiltrated corrosive media, leading to fluorescence quenching and loss of corrosion detection function. Therefore, the fluorescent probes in the coatings lack an early protection mechanism. In order to avoid the interference of the complex environment in the coatings, fluorescent probes should be loaded or encapsulated by micro-/nanocontainers, such as mesoporous nanoparticles, supramolecules or microcapsules. The loading and packaging treatment of fluorescent probes also increases the local concentration of fluorescent probes and raises the intensity of fluorescent signals in corrosion regions [16].

Zeolites (ZEO) are aluminosilicate minerals with a framework structure composed of silica tetrahedrons and alumina tetrahedrons [17]. Their molecular formula is expressed as ${\left({\mathrm{Na}}^{+},K^{+}\right)}_{x}{\left({\mathrm{Ca}}^{2+},{\mathrm{Sr}}^{2+},{\mathrm{Ba}}^{2+},{\mathrm{Mg}}^{2+}\right)}_{y}\cdot \left[{\mathrm{Al}}_{x}O_{2n}+2{\mathrm{ySi}}_{n}\left(x+2y\right)O_{2n}\right]\cdot {\mathrm{mH}}_{2}O$. The framework structure of zeolite is electronegative and balanced by metal cations (such as K+, Na^+^, Ca^2+^) [18]. Therefore, zeolites generally display cation exchange properties. They are excellent natural molecular containers with hydrothermal stability and acid resistance [19]. The pore structure of zeolites is uniform, and the specific surface area and pore volume are large. These features make them capable of adsorbing organic molecules containing polar groups (such as -NH_2_\-OH\-COOH) [20]. Due to their special structure and performance, zeolites are used to achieve adsorption, ion exchange, and also as molecular sieves [21,22].

In this paper, RBA which responds to proton-${\mathrm{Fe}}^{3+}$ synergistically, was prepared by a newly improved methods and used as a fluorescent switch probe for corrosion detection. The zeolite-supported RBA supramolecular system (RBA/ZEO) was constructed. Based on RBA/ZEO, highly sensitive and accurate fluorescence detection of local corrosion under the coatings was realized. Simultaneously, ZEO provided effective protection for RBA. The exchange and enrichment of ${\mathrm{Fe}}^{3+}$ by ZEO enhanced the fluorescence brightness. In addition, ZEO achieved the slow release of surface-loaded RBA. In this study, the proton-${\mathrm{Fe}}^{3+}$ coordinated responsive fluorescence behavior of RBA was investigated through ultraviolet-visible absorption spectroscopy and fluorescence analysis. Based on XRD, infrared spectroscopy and elemental analysis, the loading behavior of ZEO towards RBA was analyzed. Methods such as correlation spectroscopy and total organic carbon detection (TOC) were applied to study the sustained release behavior of RBA from ZEO. Finally, the effect of RBA/ZEO on the anti-corrosion performance of some coatings was evaluated from the perspective of electrochemical impedance spectroscopy.

## 2. Materials and Experiments

### 2.1. Preparation of RBA and RBA/ZEO

In previous studies, RBA was prepared by complex methods. Under the action of anhydrous hydrazine, the acyl chloride formed by rhodamine B and phosphorus oxychloride was transformed into rhodamine B hydrazide. Furthermore, the nucleophilic reaction with acetone promoted the formation of RBA. It was difficult to control the conditions of this method. Its low yield and high failure rate limited its application. Here, a new simpler preparation method is proposed: RB (2 g) was dissolved in absolute ethanol (50 mL), and stirred rapidly at room temperature. When the RB was evenly dispersed, excess anhydrous hydrazine was added dropwise to the solution. Then the solution was subjected to reflux treatment under stirring conditions for 8 h. The pink solution turned orange and a yellow precipitate was produced. The reaction was terminated and the mixture cooled to room temperature. The organic solvent was removed by filtration under reduced pressure. The products were dissolved in deionized water, and the pH was adjusted to 8~11 with 0.5 $\mathrm{mol}\cdot L^{-1}$ NaOH solution. After filtering and drying again, off-white solid RBA was obtained.

The 13X zeolite used (Aladdin Co., Shanghai, China) was spherical, with a diameter of 3~5 mm and a pore size of about 10~20 A. Its molecular formula was ${\mathrm{Na}}_{86}{\left[{\mathrm{AlO}}_{2}\right]}_{86}{\left({\mathrm{SiO}}_{2}\right)}_{106}$. After the zeolite particles were ground and screened (200 mesh), a zeolite powder was obtained. The zeolite powder was placed in a MM200 mixed ball mill (RESTCH Co., Berlin, Germany) and treated at the frequency of 20 Hz for 2 min. The surface defects of the treated zeolite increased, which improved its adsorption and loading capacity. Zeolite powder (10 g) was added to a water and acetonitrile (volume ratio 1:1) solution of $0.5\;\mathrm{mol}\cdot L^{-1}$ RBA. The mixed solution was placed in a water bath and stirred at 35 °C and 200 rpm for 24 h. After vacuum filtration, the solid materials were isolated and washed with distilled water. The products were placed into a drying oven and dried at 38 °C, and then placed in a sealed reagent bottle.

### 2.2. Characterization of RBA and RBA/ZEO

The IR of RB and RBA were detected on a Nicolet iS10 Fourier Transform Infrared (FT-IR) spectrometer (Thermo Fisher Scientific Co., Waltham, MA, USA). An inVia confocal Raman spectrometer (Renishaw Co., London, UK) was applied to measure the Raman spectra of RB and RBA. XRD pattern detection was performed on an Ultima IV X-ray diffractometer (Rigaku Co., Tokyo, Japan). The RB or RBA were ultrasonically dispersed in the mixed solution of acetonitrile and water (volume ratio of 1:1), and the spectrum was detected by a UV7600 dual-beam ultraviolet-visible spectrometer (Lengguang Co., Shanghai, China).

SEM (S-4300, Hitachi Co., Tokyo, Japan) was adopted to analyze the surface morphology of ZEO and RBA/ZEO. The elemental composition of ZEO and RBA/ZEO was characterized by the built-in EDS. Elemental analysis (EA3000, Euro Vector Co., Pavia, Italy) was selected for more precise analysis of the components of ZEO and RBA/ZEO. The XRD patterns of ZEO, RBA and RBA/ZEO were investigated on the X-ray diffractometer (Ultima IV, Rigaku Co., Tokyo, Japan). X-ray photoelectron spectroscopy (ESCALAB 250Xi, Thermo Fisher Scientific Co., Waltham, MA, USA) was used to analyze the O element in ZEO and RBA/LDHs.

### 2.3. UV-Vis Absorption and Fluorescence Behavior

RB and RBA were dispersed in acetonitrile/water solution with volume ratio of 1:1. HCl and NaOH solutions were used to adjust the proton equivalent concentration of the solution to 0~1.0 eq., that is, the multiple of proton concentration relative to RB or RBA concentration. ${\mathrm{FeCl}}_{3}$ solution was applied to adjust the ${\mathrm{Fe}}^{3+}$ equivalent concentration of the solution to 0~10 eq., that is, the multiple of ${\mathrm{Fe}}^{3+}$ concentration relative to RB or RBA concentration. The UV-vis absorption spectra of RB and RBA in varied solutions were measured. Furthermore, a Lengguang F98 fluorescence spectrometer was applied to detect the corresponding fluorescence emission spectra. The fluorescence photos were taken on a WFH-203C UV analyzer (ChiTang, Shanghai, China).

### 2.4. Release Behavior of RBA from RBA/ZEO

An appropriate amount of RBA/ZEO was dispersed in 3.5% NaCl solution and the solution samples were centrifuged for varied times. The supernatant was take to determine the total organic carbon (TOC) using a Vario TOC Cube (Elementar Co., Frankfurt, Germany). Based on the TOC data, the release behavior of RBA was further analyzed. A proper amount of RBA/ZEO was dispersed in water/acetonitrile (volume ratio 1:1) solution of $3.5\%\;\mathrm{NaCl}+500\;\mu \mathrm{mol}\;{\mathrm{Fe}}^{3+}$. A time-resolved confocal fluorescence microscopy system (MicroTime 200, PicoQuant, Berlin, Germany) was adopted to measure the fluorescence correlation spectra of the abovementioned mixed solution. The release information of RBA from ZEO was obtained based on the fitting analysis of the data.

### 2.5. Fluorescence Detection of RBA/ZEO/Epoxy Coatings

An appropriate amount of RBA/ZEO was doped into epoxy resin, and after mixing with curing agents, coatings with a thickness of 50 μm were prepared on the surface of carbon steel electrodes. X-shaped artificial scratches were made on the coating surface with a knife. The defective coating specimens were immersed in 3.5 wt.% NaCl solution for 24 h. The artificial defect free coatings were anodic polarized in 3.5 wt.% NaCl solution and +100 mV vs. OCP (open circuit potential) for 5 min to accelerate corrosion. After immersion or polarization, the samples were observed and photographed by epi-illumination fluorescence microscope.

### 2.6. Anticorrosion Performance of RBA/ZEO/Epoxy Coatings

The electrochemical measurement experiments were carried out on a Autolab PGSTAT302N electrochemical workstation (Metrohm Co., Berne, Switzerland) and a three-electrode system was adopted. The coating/steel samples were the working electrodes. A platinum sheet electrode and a saturated calomel electrode served as auxiliary electrode and reference electrode, respectively. The electrochemical impedance spectroscopy measurements were carried out in 3.5 wt.% NaCl solution. The frequency range of the AC disturbance signal is 10 mHz~100 kHz. The obtained electrochemical impedance spectra were fitted and analyzed by Zview software (Scribner Associates Inc., Southern Pines, NC, USA).

## 3. Results and Discussion

### 3.1. Structural Analysis of RBA

**Figure 1:**
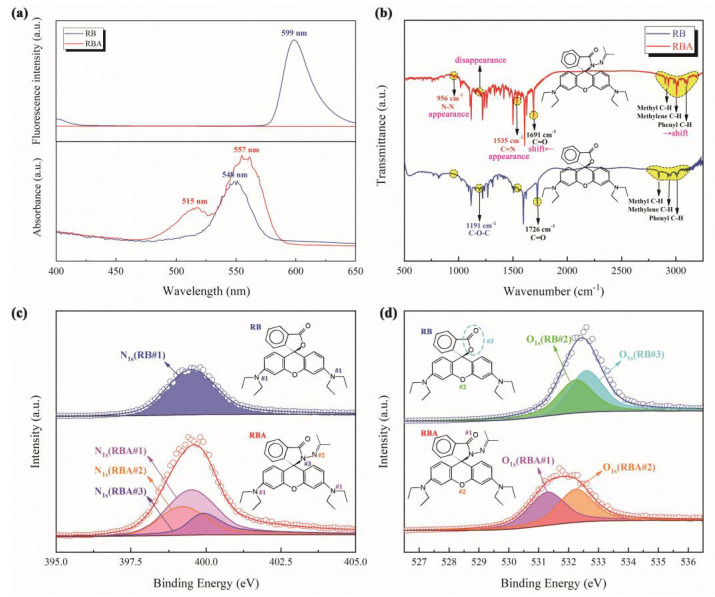
Structural analysis of RB and RBA, (**a**) UV-Vis absorption spectrum and fluorescence spectrum, (**b**) infrared spectrum, (**c**) XPS of N element and (**d**) XPS of O element.

In order to verify the successful preparation of RBA by the improved method, a variety of light spectrum and energy spectrum analyses were implemented (Figure 1). Firstly, RBA and RB exhibited different UV-Vis absorption characteristics. Due to the change in molecular structure, RBA strongly absorbed visible light at 515 nm and 557 nm, respectively. However, RB only showed one absorption peak at 548 nm. RB molecules exhibited significant fluorescence activity and emitted orange-red fluorescence with the wavelength of 599 nm. The fluorescence activity of RBA molecules was extremely weak, and they hardly emitted any fluorescence. Its fluorescence spectrum appeared as a horizontal line, as shown in Figure 1a. The infrared spectrum reflected more molecular structure information, as shown in Figure 1b. Due to the similarity of the molecular skeletons of RB and RBA, their infrared spectra were generally similar, with some local differences. In the infrared spectrum of RBA, characteristic peaks corresponding to N-N and C=N bonds appeared, while the characteristic peaks of C-O-C appearing in the IR spectrum of RB disappeared. This meant that the lactone structure in the RB molecule was destroyed and the acylhydrazone group was formed. Affected by the functional group changes, the characteristic C=O and C-H peaks shifted accordingly. In order to further corroborate the presence of the acylhydrazone structure in the RBA molecule, XPS of N and O were measured. In Figure 1c, only one type of N atom exists in the RB molecule, while the XPS of N in RBA consisted of three peaks, which corresponded to three different types of N atoms in the RBA molecule. There were also differences in the O1s XPS of the two molecules, as shown in Figure 1d. The O1s XPS of the RB molecule was divided into two peaks, which represented the O atoms in the ester group and ether structure. The binding energy of the two O atoms in the ester group was too close to distinguish. After the acylhydrazone structure was formed, the XPS of O shifted to the left as a whole. After peak splitting, the O1s XPS of the ether structure was basically unchanged, and the binding energy of O in acylhydrazone group appeared at about 531.4 eV. In short, the light spectrum and energy spectrum results verified the successful synthesis of RBA.

### 3.2. Loading Behavior and Characterization of ZEO/RBA

**Figure 2:**
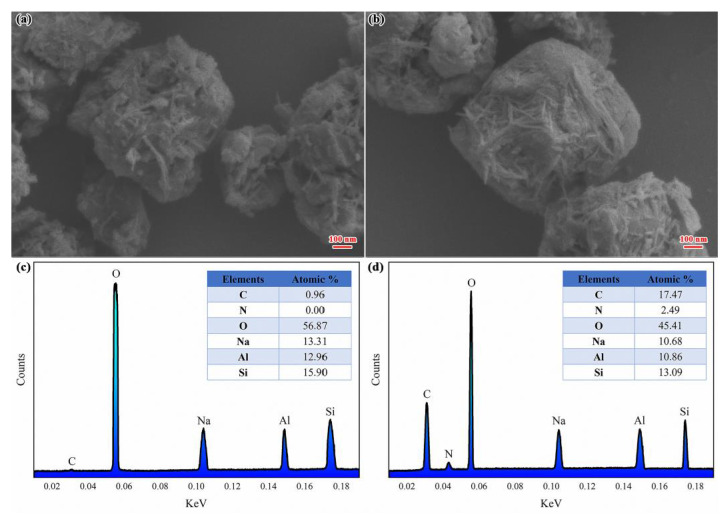
SEM photos of (**a**) ZEO and (**b**) RBA/ZEO and energy dispersive spectroscopy (EDS) of C, N, O, Na, Al, Si in (**c**) ZEO and (**d**) RBA/ZEO.

Figure 2 shows SEM photos of ZEO and RBA/ZEO. The size of the ZEO and RBA/ZEO particles was about 800 nm~1 μm. After loading RBA, the morphology and structure of ZEO did not changed significantly. This was because zeolites mainly adsorb organic molecules into the interior or surface through physical effects such as dispersion forces and electrostatic attraction. Therefore, the structure of the zeolite was not transformed. According to the EDS results, the main elements of the zeolite sample were O, Na, Al and Si, and the N element was not included, as shown in Figure 2c. This was consistent with the expectation. Figure 2d shows that N atoms existed in RBA/ZEO and they originated from the RBA molecules.

**Figure 3:**
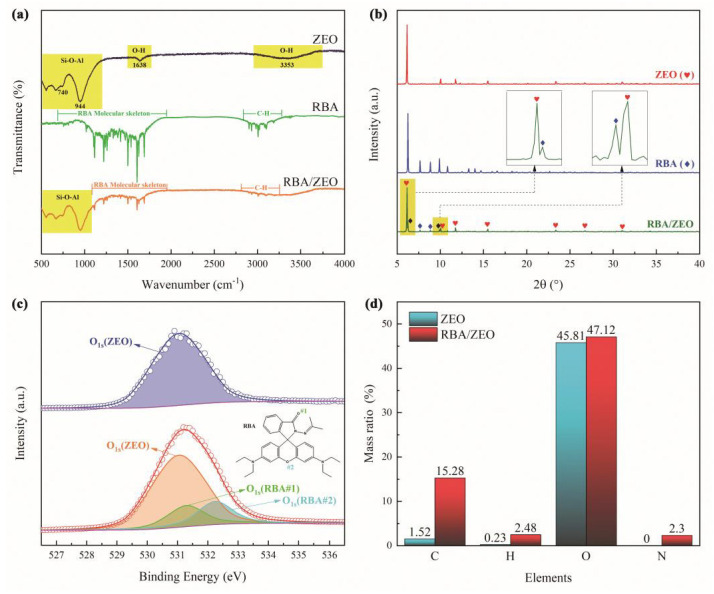
Structure and composition characterization of ZEO and RBA/ZEO, (**a**) infrared spectrum, (**b**) XRD, (**c**) XPS of O element and (**d**) element analysis.

In order to further verify the loading of RBA by ZEO, infrared spectroscopy detections were implemented, as shown in Figure 3a. The infrared spectrum of ZEO showed the characteristic bands of the Si-O-Al structures and O-H bonds. In the infrared spectrum of RBA, the absorption band in range of $700{\;\mathrm{cm}}^{-1}\sim 1800{\;\mathrm{cm}}^{-1}$ reflected the molecular skeleton of RBA, and the absorption band in the range of $2800{\;\mathrm{cm}}^{-1}\sim 3200{\;\mathrm{cm}}^{-1}$ corresponded to the vibration of C-H bonds. The infrared spectrum of RBA/ZEO included both the Si-O-Al absorption band of ZEO and the molecular skeleton absorption band of RBA. In addition, Figure 3b illustrates that RBA/ZEO exhibited the X-ray diffraction characteristics of RBA and ZEO, which demonstrated the loading of RBA by ZEO on the other hand. In the O1s XPS shown in Figure 3c, the O1s peak of ZEO corresponds to the oxygen atoms in the Si-O-Al structure. The O1s XPS of RBA/ZEO was divided into three peaks. Aside from the O1s peak of ZEO, the other two peaks belonged to the ether structure and acylhydrazone group in the RBA molecule. The quantitative calculation of RBA load ratio was realized from the elemental analysis data. Figure 3d displays the elemental analysis results of ZEO and RBA/ZEO. Due to the loading of RBA, the contents of C, H and N in RBA/ZEO were significantly increased. In order to avoid the influence of water, carbon dioxide in the air and impurities in ZEO, and due to the fact there was no N element in ZEO, the load ratio of RBA was calculated based on the N element using the following formula.

(1) $$L_{\mathrm{RBA}}=\frac{\left(N_{\mathrm{RBA}/\mathrm{ZEO}}\%-N_{\mathrm{ZEO}}\%\right)\times M_{\mathrm{RBA}}}{M_{N}\times {\mathrm{Num}}_{N}}\times 100\%$$

where, $L_{\mathrm{RBA}}$ was the load ratio of RBA, and $N_{\mathrm{ZEO}}\%$ and $N_{\mathrm{RBA}/\mathrm{ZEO}}\%$ were the mass ratios of N elements in ZEO and RBA/ZEO, respectively. $M_{N}$ and $M_{\mathrm{RBA}}$ were the relative atomic/molecular masses of N and RBA, respectively. ${\mathrm{Num}}_{N}$ was the number of N atoms in the RBA molecule.

In the same way, the load ratio of RBA based on the data of C, H and O elements was calculated and presented in Table 1. Due to the influence of carbon dioxide and impurities, the results based on C and H elements differed greatly from that of N element. In this study, the results derived from the N element were adopted.

### 3.3. Anti-Corrosion Performance of RBA/ZEO Coatings

**Figure 4:**
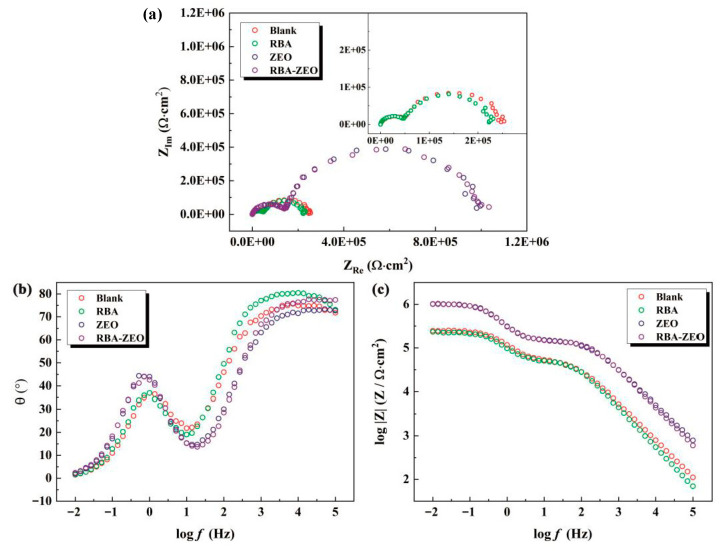
Electrochemical impedance spectroscopy of four epoxy coatings, (**a**) Nyquist, (**b**) Bode-phase angle and (**c**) Bode-|Z|.

Fluorescence detection of corrosion located under the coatings and at defects is the additional function of anti-corrosion coatings. The additional function should not weaken the main function of the coatings, that is, corrosion protection. Therefore, epoxy coatings, RBA/epoxy coatings, ZEO/epoxy coatings and RBA/ZEO/epoxy coatings were prepared, and electrochemical impedance spectroscopy was chosen to investigate the anti-corrosion performance of the coatings to clarify the influence of fluorescent functional fillers. Figure 4 shows the electrochemical impedance spectra of the four epoxy coatings. The Nyquist diagrams of the epoxy coatings and RBA/epoxy coatings were similar, and the values of ${\left|Z\right|}_{f=0.01}$ were close. The Nyquist diagrams of ZEO/epoxy coatings and RBA/ZEO/epoxy coatings were similar, and the values of ${\left|Z\right|}_{f=0.01}$ were almost equal. This implied that the presence of fluorescent probe RBA had little effect on the anti-corrosion properties of the coatings. It also verified that the physical adsorption of RBA had little effect on the structure and performance of ZEO, which was consistent with the conclusion from the SEM data in Figure 2. In addition, Nyquist diagrams showed that the capacitive reactance arcs of ZEO-containing coatings were significantly greater than that of ZEO-free coatings. Correspondingly, the ${\left|Z\right|}_{f=0.01}$ values of the ZEO-containing coatings were about 0.6 orders of magnitude higher than that of the ZEO-free coatings.

**Figure 5:**
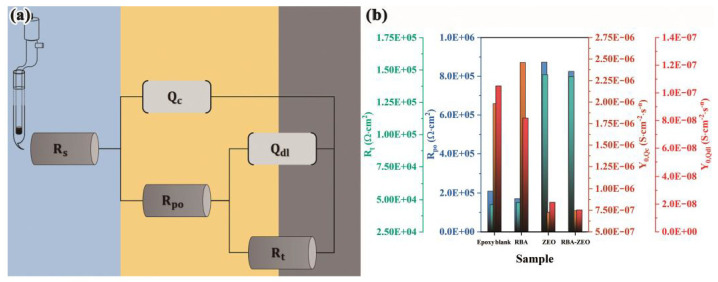
The (**a**) equivalent circuit for the fitting of electrochemical impedance spectroscopy and (**b**) the fitting results.

The Nyquist diagrams of the four coatings all exhibited two capacitive reactance arcs, which represented two time-constants and two electrode processes of the coating/steel sample. Correspondingly, the Bode-phase angle diagrams presented one ‘half peak’ at the high frequency end, and there was one peak near logf≈0. Theoretically, the ‘half peak’ would form a complete peak in the higher frequency range. Therefore, the characteristics of the phase angle coincided with the two time-constants. According to the analysis of the Nyquist and phase angle, the equivalent circuit shown in Figure 5a was established. $R_{s}$ is the solution resistance, which represents the resistance to ion migration between the reference electrode and the sample in the solution. $Q_{c}$ is a constant phase angle element representing the capacitance of the coatings. Coating capacitance is the capacitance between solution/coating interface and metal/coating interface. The constant phase angle element included the deviation of the actual capacitance from the ideal capacitance. $R_{\mathrm{po}}$ is the coating micropore resistance, which represents the resistance of the electrolyte solution penetrating into the micropores of the coatings. Therefore, $R_{\mathrm{po}}$ reflects the path length of micropores in the coatings and the diffusion resistance of corrosive media. $Q_{\mathrm{dl}}$ is a constant phase angle element representing the electric double layer capacitance. $R_{t}$ is the charge transfer resistance of the electrochemical oxidation of the metal. The fitting results based on the equivalent circuit are shown in Figure 5b and Table 2. The presence of RBA exhibited a weak effect on the parameters of the electrical components of the epoxy blank coatings and ZEO/epoxy coatings. The addition of ZEO and RBA/ZEO significantly improved the coating micropore resistance $R_{\mathrm{po}}$. This is because nano-scale ZEO (or RBA/ZEO) fills the micropores such as pinholes and defects of the coatings, and increases the difficulty of diffusion and penetration of corrosive media into the coatings. The capacitance of the coatings is positively related to water content, so the admittance coefficient $Y_{0,Q_{c}}$ of the coating capacitance dropped sharply with the addition of ZEO or RBA/ZEO. This indicates an enhancement of the anti-corrosion performance. Therefore, the two parameters $Q_{\mathrm{dl}}$ and $R_{t}$, which reflect the electrochemical corrosion process of metals, changed significantly. The increase of $R_{t}$ indicated that the resistance of metal oxidation raised. $Q_{\mathrm{dl}}$ is related to the area of metal/solution interface, and the decrease of its admittance coefficient $Y_{0,Q_{\mathrm{dl}}}$ reflects the reduction of the scale of corrosion. In short, the application of RBA/ZEO functional filler not only did not weaken the main anti-corrosion performance of the coatings, but also significantly improved it.

### 3.4. Corrosion Factor Responsive Fluorescence Switching Behavior of RBA

**Figure 6:**
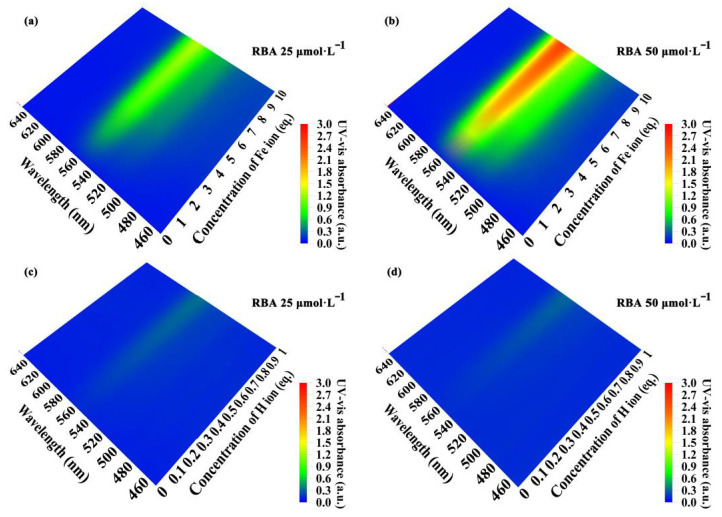
The ultraviolet-visible absorption behavior of RBA response to iron ion and proton, (**a**) $\mathrm{RBA}\;25\;\mu \mathrm{mol}\cdot L^{-1},{\;\mathrm{Fe}}^{3+}$, (**b**) $\mathrm{RBA}\;50\;\mu \mathrm{mol}\cdot L^{-1},{\;\mathrm{Fe}}^{3+}$, (**c**) $\mathrm{RBA}\;25\;\mu \mathrm{mol}\cdot L^{-1},{\;H}^{+}$, (**d**) $\mathrm{RBA}\;50\;\mu \mathrm{mol}\cdot L^{-1},{\;H}^{2+}$.

Since the fluorescent functional filler had no negative impact on the anti-corrosion performance of the coatings, the corrosion detection ability was studied next. The important features of localized corrosion that occurred under the coatings and in defects were the production of metal ions and local acidification. Therefore, the iron ion and proton sensitive fluorescence of RBA were the prerequisites for detecting steel corrosion. To realize the emission of fluorescence, the fluorescent molecules must first be excited by photons. The UV-Vis spectra of RBA in different concentrations of iron ions and protons were measured (Figure 6). The iron ion concentration was expressed in equivalents, and its meaning is the multiple of the RBA concentration. For example, for RBA of $25\;\mu \mathrm{mol}\cdot L^{-1}$, the equivalent value of ${\mathrm{Fe}}^{3+}$ 5 eq. represented concentrations of $25\times 5=125\;\mu \mathrm{mol}\cdot L^{-1}$. The proton concentration was described in a similar way. In the environment without iron ions, RBA did not exhibit any UV-visible absorption peaks. When RBA and iron ions formed the complex, the UV-Vis absorption peaks at about 560 nm were gradually strengthened with the increase of iron ion concentration, as shown in Figure 6a,b. Similarly, RBA presented a weak absorption of light in a neutral environment. The absorbance increased with the acidification of the solution, as shown in Figure 6c,d. In addition, the absorbance and the concentration of RBA obviously showed a positive correlation.

**Figure 7:**
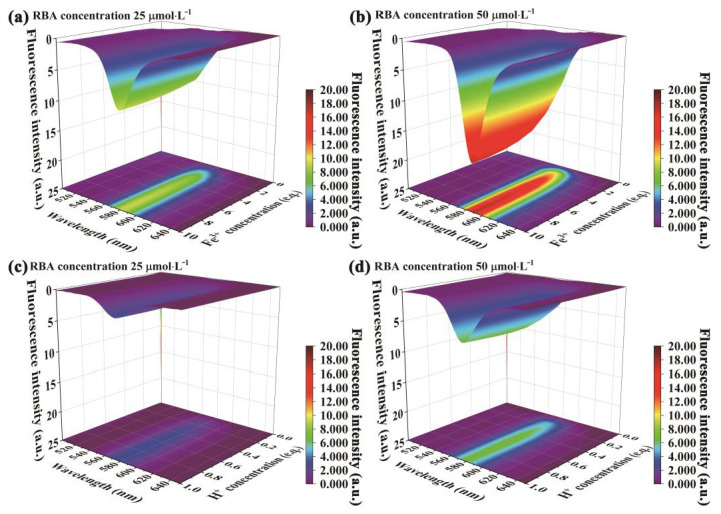
Fluorescence emission behavior of RBA response to iron ion and proton, (**a**) $\mathrm{RBA}\;25\;\;\mu \mathrm{mol}\cdot L^{-1},{\;\mathrm{Fe}}^{3+}$, (**b**) $\mathrm{RBA}\;50\;\mu \mathrm{mol}\cdot L^{-1},{\;\mathrm{Fe}}^{3+}$, (**c**) $\mathrm{RBA}\;25\;\mu \mathrm{mol}\cdot L^{-1},{\;H}^{+}$, (**d**) $\mathrm{RBA}\;50\;\mu \mathrm{mol}\cdot L^{-1},{\;H}^{2+}$.

**Figure 8:**
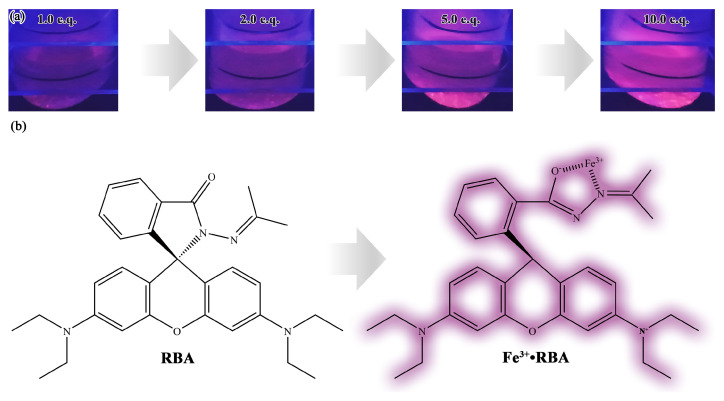
The (**a**) fluorescence photos of RBA in iron ion environments differed in concentrations and (**b**) the complexation reaction mechanism of ${\mathrm{Fe}}^{3+}$ activated fluorescence.

**Figure 9:**
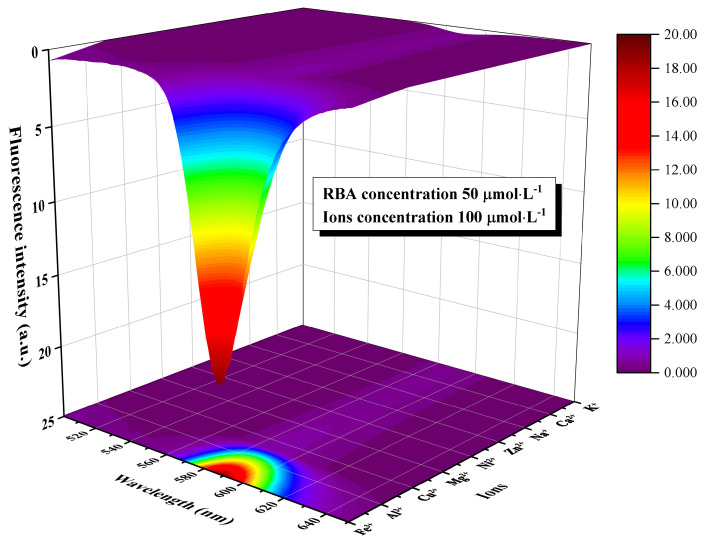
The integrated fluorescence intensity of RBA in different metal ions environments which were equal in concentration.

The fluorescence emission spectra of RBA in an iron ion or proton-rich environments that differed in concentration are shown in Figure 7. In the environment without iron ions, RBA basically did not exhibit any fluorescence activity. When RBA and iron ions formed the complex, fluorescence emission peaks gradually appeared in the interval between 575 and 585 nm, and were strengthened with the increase of iron ion concentration, as shown in Figure 7a,b. The photos in Figure 8a intuitively show that the fluorescence was enhanced by iron ions. The enhancement was attributed to the formation of complexes, which transform the molecular structure of RBA and realize the fluorescence switch function, as shown in Figure 8b. Similarly, RBA did not emit fluoresce in a neutral environment. The greater the acidity of the solution, the stronger the fluorescence intensity, as shown in Figure 7c,d. The strengthening tendency of fluorescence intensity gradually slowed down with the increase of iron ion or proton concentration. This was due to the raise in concentration of iron ions or protons, which raised the concentration of active fluorescent molecules, and in turn promoted the probability of molecular collisions that led to more vibrational relaxation processes. In addition, the fluorescence intensity and the concentration of RBA obviously exhibited a positive correlation. In order to investigate the selectivity of RBA to iron ions, the integrated fluorescence intensity of RBA in different metal ion environments (equal in concentration) were measured, as shown in Figure 9. The RBA displayed a very weak fluorescence response to other metal ions. Therefore, its iron ion selectivity was extremely significant. This is of great significance to resist the interference of impurity ions and ensure the accuracy of corrosion detection.

### 3.5. Release Behavior of RBA/ZEO

Fluorescence correlation spectroscopy is a kind of single molecule detection technology. It records information about fluorescence intensity fluctuations caused by the diffusion of fluorescent molecules in the confocal observation volume. The confocal observation volume was ellipsoidal, and in this experiment, its axial radius was z=1.0 μm, and the radial radius was r=0.2 μm. The auto-correlation function G(τ) of the fluorescence fluctuation curve was obtained by the following equation:

(2) $$G\left(\tau \right)=\frac{\langle \delta I\left(t+\tau \right)\delta I\left(t\right)\rangle }{\langle I{\left(t\right)}^{2}\rangle }$$

**Figure 10:**
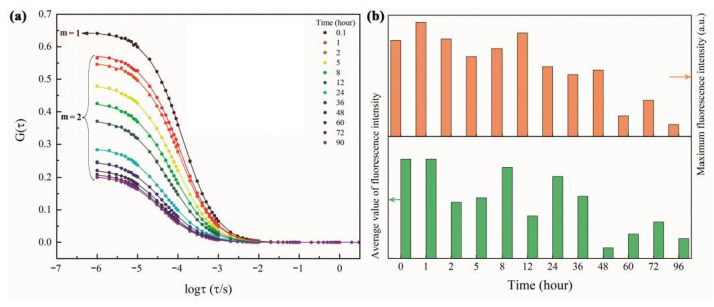
The (**a**) fluorescence correlation spectra and fluorescence fluctuation curves (inset) of RBA/ZEO in $3.5\%\;\mathrm{NaCl}+500\;\mu \mathrm{mol}\;{\mathrm{Fe}}^{3+}$ solution and (**b**) the statistical information of fluorescence fluctuation data.

The auto-correlation functions possessed the following two specific forms, respectively:

(3) $$G\left(\tau \right)=\overset{m}{\underset{i=1}{\sum }}\frac{1}{N}\frac{f_{i}}{\left(1+\frac{\tau }{\tau_{D}}\right)\sqrt{1+\frac{\tau }{s^{2}\tau_{D}}}}$$

where, *N* is the average number of fluorescent molecules in the observed volume, and $\tau_{D}$ is the duration of fluorescence molecules diffused through the observed volume. They are obtained through fitting the fluorescence correlation spectroscopy by the auto-correlation function G(τ). s is the ratio of axial radius $z_{0}$ to radial radius $r_{0}$ of the observed volume, and $f_{i}$ is the ratio of fluorescence molecule i. Figure 10 show the fluorescence fluctuation curves and fluorescence correlation spectroscopy of RBA/ZEO in a $3.5\%\;\mathrm{NaCl}+500\;\mu \mathrm{mol}\;{\mathrm{Fe}}^{3+}$ environment.

**Figure 11:**
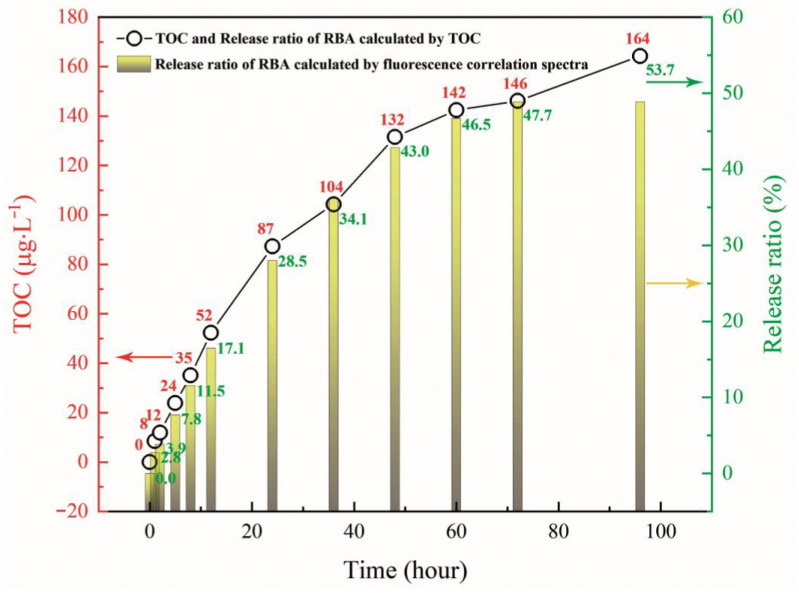
TOC detection results of RBA/ZEO dispersed in 3.5% NaCl solution for different times and RBA release ratio calculated from TOC (dot and line) and fluorescence correlation spectra (histogram).

The initial fluorescence correlation spectrum (t=0.1 h) was fitted by the autocorrelation function of m=1. This meant that there was only one type of fluorescent particle (or molecule) in the solution. The fitting results showed $\tau_{D}=2.98\times 10^{-2}\;s$ for this fluorescent particle. According to the Einstein diffusion (Equation (4)) and the Stokes-Einstein relationship (5), the diffusion rate coefficient of the fluorescent particles is $D=3.36\times 10^{-13}{\;m}^{2}\cdot s^{-1}$ and hydrodynamic radius is $R_{h}\approx 1000\;\mathrm{nm}$:

(4) $$\tau_{D}=\frac{r^{2}}{D}$$

(5) $$R_{h}=\frac{\kappa_{B}T}{6\pi \eta D}$$

where, $\kappa_{B}$ is Boltzmann constant, T is the thermodynamic temperature, and η is the viscosity coefficient of the solution. According to the hydrodynamic radius, the fluorescent particles were judged to be RBA/ZEO particles. The fitting of subsequent fluorescence correlation spectra (t=1~90 h) required m=2 in the autocorrelation function G(τ), that is, there were two types of fluorescent particles (or molecules) in the system. Their $\tau_{D}$ were $\tau_{D1}=2.87\times 10^{-2}\;s$ and $\tau_{D2}=3.28\times 10^{-5}\;s$, respectively. Correspondingly, $R_{h1}\approx 1000\;\mathrm{nm}$ and $R_{h2}\approx 1.1\;\mathrm{nm}$. This indicated that the RBA molecules were released from the ZEO carrier. Similarly, the concentrations of RBA and RBA/ZEO at each time point were estimated according to Equation (6) and the fitting results of N and $f_{i}$:

(6) $$c=\frac{{\mathrm{Nf}}_{i}}{\frac{4}{3}N_{A}{\pi r}^{2}z}$$

where, $N_{A}$ is Avogadro’s constant. The calculation of concentration facilitated further investigation of the release ratio and release behavior of RBA. According to Equation (7), the release ratio of RBA ($R_{\mathrm{RBA}}$) was obtained:

(7) $$R_{\mathrm{RBA}}=\frac{{\mathrm{Nf}}_{i}}{\frac{4}{3}N_{A}{\pi r}^{2}{\mathrm{zmL}}_{\mathrm{RBA}}}\times 100\%$$

where m is the mass concentration of the test solution. The results are shown in the histogram of Figure 11.

**Figure 12:**
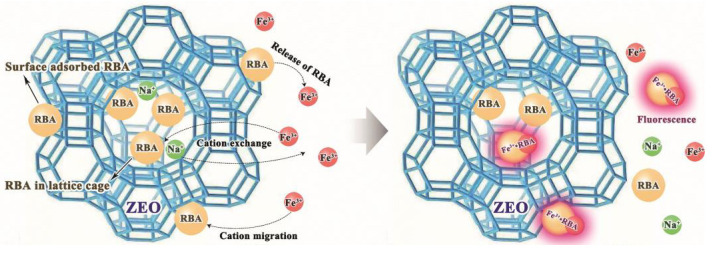
The mechanism of the loading, release and fluorescence activity of RBA.

Figure 11 includes the total organic carbon test results and the RBA release ratio ($R_{\mathrm{RBA}}^{\prime }$) calculated by TOC (dotted line graph). The calculation method was:

(8) $$R_{\mathrm{RBA}}^{\prime }=\frac{\mathrm{TOC}\cdot \frac{M_{\mathrm{RBA}}}{n_{C}M_{C}}}{{\mathrm{mL}}_{\mathrm{RBA}}}\times 100\%\;$$

where, $M_{\mathrm{RBA}}$ is the molecular weight of RBA, $n_{C}$ is the number of carbon atoms in RBA molecule, and $M_{C}$ is the atomic weight of carbon. The $R_{\mathrm{RBA}}$ values obtained by the two methods were basically the same in terms of value and evolution trend. The early release rate of RBA was faster, and then slowed down. $R_{\mathrm{RBA}}$ tended to be level in the later stage, which illustrated that RBA was basically no longer being released. This was because the load of RBA on ZEO was divided into two types. The first was the RBA contained in the lattice cage of ZEO, and the second was the RBA was adsorbed on the surface of ZEO. The former was difficult to be released, and the released RBA mainly originated from the latter. In the analysis of fluorescence correlation spectroscopy, there were two kinds of luminescent particles, RBA and RBA/ZEO, in the solution. The indicated that the RBA adsorbed on the surface of ZEO was released into the solution and emitted fluorescence after complexing with iron ions. At the same time, the RBA in the ZEO lattice cage could also contact with iron ions and formed complexes, leading to fluorescent behavior. This was due to the cation exchange of ZEO, and the iron ions migrating into the lattice cage of ZEO, as shown in Figure 12. The fluorescence behavior of the released RBA was inevitably disturbed by the complex corrosive environment. Therefore, with the passage of time and the release of RBA, the maximum and average fluorescence intensity decreased, as shown in Figure 10b. This implies that the fluorescence system was more suitable for early corrosion detection, which was in line with our design expectations.

### 3.6. Corrosion Detection Performance of RBA/ZEO in Coatings

**Figure 13:**
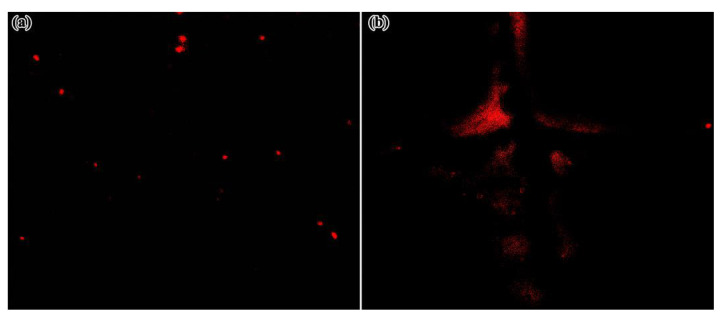
Corrosion detection performance of RBA/ZEO/epoxy coatings, (**a**) coating sample of accelerated corrosion by polarization and (**b**) defective coating with X-shaped scratch.

The corrosion detection performance of RBA/ZEO in coatings was investigated. Figure 13 shows tfluorescence photos of two kinds of coatings containing RBA/ZEO. The corrosion of the coated sample in Figure 11a was accelerated by polarization. Due to the inhomogeneity of the microstructure of the metal surface, polarization would not cause uniform corrosion. Crystal defects, geometric defects and impurity sites on the metal surface preferentially corroded, forming pitting corrosion randomly scattered on the metal surface. The multiple randomly distributed bright spots in the fluorescence photo indicated the location of pitting corrosion after accelerated corrosion. For coated samples with X-shaped scratches, corrosion spread from the intersection of the scratches to the surroundings. This corresponds to the light-emitting area in Figure 11. Simultaneously, the edge of the scratch was also fluorescent due to corrosion. It could be seen from the fluorescence photos that the RBA/ZEO in the coatings effectively indicated the occurrence of corrosion under the coatings and accurately located the corrosion site.

## 4. Conclusions

In this paper, RBA and zeolite supported RBA (RBA/ZEO) were successfully prepared. The coordination of fluorescent-inactive RBA and ${\mathrm{Fe}\;}^{3+}$ formed fluorescent-active ${\mathrm{Fe}\;}^{3+}\cdot \mathrm{RBA}$ complex, which realized a ${\mathrm{Fe}\;}^{3+}$ responsive fluorescent on-off behavior. The results showed that the higher the ${\mathrm{Fe}\;}^{3+}$ content in the solution, the more obvious the UV-Vis absorption and the higher the fluorescence intensity. This was due to the transition from the spirolactam structure of RBA to the ring opened amide structure of the complex. Protonation also turned on the fluorescence activity of RBA. The formation of ${\mathrm{Fe}\;}^{3+}$ and anodic acidification were the characteristics of local corrosion. The ${\mathrm{Fe}\;}^{3+}$ and proton responsive fluorescence of RBA established the theoretical foundation for corrosion detection. The RBA/ZEO filled in the epoxy coatings effectively indicated the occurrence of corrosion under the coatings and at defects, and accurately located the corrosion sites. RBA was protected by the loaded in/on ZEO. This protected RBA from fluorescence quenching due to premature exposure to the corrosive environment. There were two types of loading: surface adsorption and loading in lattice cages. The former easily released RBA. The cation exchangeability of ZEO enabled iron ions to complex with RBA in the lattice cage and produced the fluorescence activity of RBA/ZEO. When RBA/ZEO were applied in the coatings, it improved the shielding performance of the coatings due to the filling effect of ZEO on the micropores of the coatings. Simultaneously, it indicated the occurrence of corrosion and located the corrosion sites and coating defects accurately in the form of fluorescence.

## Figures and Tables

**Table 1:** RBA load ratios (%) derived from N, C, H and O elements.

Elements	Element Mass Ratio in ZEO	Element Mass Ratio in RBA/ZEO	RBA Load Ratio
N	0	2.30	20.38
C	1.52	15.28	18.35
H	0.23	2.48	31.00
O	45.81	47.12	20.31

**Table 2:** The fitting results of electrochemical impedance spectroscopy.

Coatings	Epoxy Blank	RBA	ZEO	RBA/ZEO
$R_{s}\;\left(\Omega \cdot {\mathrm{cm}}^{2}\right)$	11.31	15.34	8.56	10.70
$Y_{0,Q_{c}}\;\left(\Omega^{-1}\cdot {\mathrm{cm}}^{-2}\cdot s^{-n}\right)$	1.98 × 10^−6^	2.59 × 10^−6^	7.04 × 10^−7^	7.67 × 10^−7^
$n_{Q_{c}}$	0.86	0.93	0.89	0.92
$R_{\mathrm{po}}\;\left(\Omega \cdot {\mathrm{cm}}^{2}\right)$	2.16 × 10^5^	1.83 × 10^5^	8.35 × 10^5^	8.31 × 10^5^
$Y_{0,Q_{\mathrm{dl}}}\;\left(\Omega^{-1}\cdot {\mathrm{cm}}^{-2}\cdot s^{-n}\right)$	1.18 × 10^−7^	8.66 × 10^−8^	2.24 × 10^−8^	1.59 × 10^−8^
$n_{Q_{\mathrm{dl}}}$	0.85	0.92	0.83	0.86
$R_{\mathrm{ct}}\;\left(\Omega \cdot {\mathrm{cm}}^{2}\right)$	5.05 × 10^4^	4.63 × 10^4^	1.56 × 10^5^	1.43 × 10^5^

## Data Availability

The data presented in this study are available on request from the corresponding author.
